# Development and Assessment of a Patient-Reported Outcome Instrument for Gender-Affirming Care

**DOI:** 10.1001/jamanetworkopen.2025.4708

**Published:** 2025-04-18

**Authors:** Manraj N. Kaur, Charlene Rae, Shane D. Morrison, Alexis Laungani, Pierre Brassard, Margriet G. Mullender, Tim C. van de Grift, Danny A. Young-Afat, Jens Ahm Sørensen, Lotte Poulsen, Sylvie D. Cornacchi, Jack G. Graesser, Michelle Mistry Igbokwe, Thomas Satterwhite, John H. Pang, Arya A. Akhavan, Allison Hu, Natasha Johnson, Stefan J. Cano, Kinusan Savard, Gerhard S. Mundinger, Fermín Capitán-Cañadas, Daniel Simon, Luis Capitán, Devin Coon, Hilliard T. Brydges, Rachel Bluebond-Langner, Eduardo D. Rodriguez, Lee C. Zhao, Kathleen A. Armstrong, Nicola R. Dean, Tamara A. Crittenden, Zac A. Cannell, Megan Lane, Caleb A. Haley, Jessica Hsu, Geolani W. Dy, Blair R. Peters, Jens U. Berli, Christina E. Milano, Christian X. Lava, Kenneth L. Fan, Gabriel A. Del Corral, Christodoulos Kaoutzanis, Nargis Kalia, Ty Higuchi, Oren Ganor, Sangeeta Subedi, Laura M. Douglass, Alireza Hamidian Jahromi, Helia C. Hosseini, Jacqueline Ihnat, Neil Parikh, Kevin Hu, Michael Alperovich, Edward C. Ray, Youssef Aref, Bashar A. Hassan, Fan Liang, Lily Mundy, Mang L. Chen, Andrea L. Pusic, Anne F. Klassen

**Affiliations:** 1 Patient-Reported Outcomes and Values, & Experience Center (PROVE), Brigham and Women’s Hospital, Harvard Medical School, Boston, Massachusetts; 2 Department of Pediatrics, McMaster University, Hamilton, Ontario, Canada; 3 University of Washington Medical Center, Seattle; 4 Plastic Surgery Division, GrS Montréal, Montreal, Quebec, Canada; 5 Department of Plastic Reconstructive and Hand Surgery, Amsterdam University Medical Center, Amsterdam, the Netherlands; 6 Research Unit for Plastic Surgery, University of Southern Denmark, Odense, Denmark; 7 Department of Plastic Surgery and Burns Treatment, Rigshospitalet, Copenhagen, Denmark; 8 Align Surgical Associates Inc, San Francisco, California; 9 Modus Outcomes (a division of Thread), St James House, Cheltenham, United Kingdom; 10 Fleming College, Noelville, Ontario, Canada; 11 Crane Center for Transgender Surgery, Austin, Texas; 12 The Facialteam Group, HC Marbella International Hospital, Málaga, Spain; 13 Brigham and Women’s Hospital, Harvard Medical School, Boston, Massachusetts; 14 Hansjörg Wyss Department of Plastic Surgery, NYU Grossman School of Medicine, New York, New York; 15 Department of Urology, NYU Grossman School of Medicine, New York, New York; 16 Department of Surgery, Women’s College Hospital, Toronto, Ontario, Canada; 17 College of Medicine and Public Health, Flinders University, Bedford Park, South Australia, Australia; 18 Trans Health South Australia, Level 5, Flinders Medical Centre, Bedford Park, South Australia, Australia; 19 Section of Plastic Surgery, University of Michigan, Ann Arbor; 20 Department of Urology, Oregon Health & Science University, Portland; 21 Department of Surgery, Division of Plastic and Reconstructive Surgery, Oregon Health & Science University, Portland; 22 Family Medicine, Oregon Health & Science University, Portland; 23 Department of Plastic and Reconstructive Surgery, MedStar Georgetown University Hospital, Washington, DC; 24 Department of Plastic and Reconstructive Surgery, MedStar Franklin Square Medical Center, Baltimore, Maryland; 25 Department of Surgery, Division of Plastic and Reconstructive Surgery, University of Colorado, Anschutz Medical Campus, Aurora; 26 Department of Surgery, Division of Urology, University of Colorado, Anschutz Medical Campus, Aurora; 27 Department of Plastic and Oral Surgery, Boston Children’s Hospital, Boston, Massachusetts; 28 Department of Urology, Lewis Katz School of Medicine at Temple University, Philadelphia, Pennsylvania; 29 Department of Plastic and Reconstructive Surgery, Lewis Katz School of Medicine at Temple University, Philadelphia, Pennsylvania; 30 Plastic and Reconstructive Surgery, Yale School of Medicine, New Haven, Connecticut; 31 Department of Surgery, Cedars-Sinai Medical Center, Los Angeles, California; 32 Plastic Surgery, Johns Hopkins, Baltimore, Maryland; 33 G.U. Recon, San Francisco, California; 34 Division of Plastic and Reconstructive Surgery, Brigham and Women’s Hospital, Boston, Massachusetts

## Abstract

**Importance:**

There is an urgent need for a validated gender-affirming care–specific patient-reported outcome measure (PROM).

**Objective:**

To field test the GENDER-Q, a new PROM for gender-affirming care, in a large, international sample of transgender and gender diverse (TGD) adults and evaluate its psychometric properties.

**Design, Setting, and Participants:**

This international cross-sectional study was conducted among TGD adults aged 18 years and older who were seeking or had received gender-affirming care within the past 5 years at 21 clinical sites across Canada, the United States, the Netherlands, and Spain; participants were also recruited through community groups (eg, crowdsourcing platform, social media). The study was conducted between February 2022 and March 2024. Participants had to be capable of completing the instrument in English, Danish, Dutch, or French-Canadian. Eligible participants accessed an online REDCap survey to complete sociodemographic questions and questions about gender-affirming care they had received or sought (ie, to look, function, or feel masculine, feminine, gender fluid, or another way).

**Main Outcome and Measures:**

Branching logic was used to assign relevant instrument scales. Rasch measurement theory (RMT) analysis was used to examine the fit of the observed data to the Rasch model for each scale. Test-retest reliability and hypothesis-based construct validity of instrument scales were examined. The hypothesis was that instrument scale scores would increase with better outcomes on corresponding categorical questions.

**Results:**

A total of 5497 participants (mean [SD] age, 32.8 [12.3] years; 1837 [33.4%] men; 1307 [23.8%] nonbinary individuals; and 2036 [37.0%] women) completed the field test survey. Participants sought or had the following types of gender-affirming care: 2674 (48.6%) masculinizing, 2271 (41.3%) femininizing, and 552 (10.0%) other. RMT analysis led to the development of 54 unidimensional scales and 2 checklists covering domains of health-related quality of life, sexual, urination, gender practices, voice, hair, face and neck, body, breasts, genital feminization, chest, genital masculinization, and experience of care. Test-retest reliability of the scales (intraclass correlation coefficient [average] >0.70) was demonstrated. Only 1 item (phalloplasty donor flap) had an ICC less than 0.70. As hypothesized, scores increased incrementally with better associated self-reported categorical responses. For example, among 661 participants who reported poor psychological well-being, the mean (SD) scale score was 45 (18) points; for those who reported excellent psychological well-being, the mean (SD) scale score was 85 (16) points (*P* < .001).

**Conclusions and Relevance:**

In this cross-sectional study of 5497 TGD adults, the instrument demonstrated reliability and validity. The instrument was validated in an international sample and is designed to collect and compare evidence-based outcome data for gender-affirming care from the patients’ perspective.

## Key Points

**Question:**

How does a comprehensive patient-reported outcome measure (PROM) to assess outcomes of gender-affirming care perform in an international sample?

**Findings:**

In this cross-sectional study, the instrument was developed following internationally established guidelines for PROM development, with data collected from 5497 transgender and gender-diverse adults. Psychometric analysis resulted in 54 independently functioning scales and 2 checklists; test-retest reliability and construct validity of the instrument were established.

**Meaning:**

In this study, a modular, scientifically rigorous internationally validated PROM that can be used to measure outcomes of gender-affirming care in clinical care, research, quality improvement, and regulatory efforts was developed and tested.

## Teaser

This cross-sectional study surveys transgender and gender-diverse adults to develop and test a patient-reported outcome measure for gender-affirming care.

## Introduction

Gender-affirming care encompasses social, psychological, behavioral, and medical interventions aimed at affirming gender identity and alleviating gender-related distress.^1^ Transgender and gender diverse (TGD) individuals, whose gender identity or expression differs from their sex assigned at birth, represent a growing population globally. Many seek gender-affirming care to harmonize aspects of their lives—such as appearance, emotional well-being, and social interactions—with their gender identity. This care is specialized and multidisciplinary, integrating primary, secondary, and tertiary health care services.

Health-related quality of life (HRQL) is a critical measure of the quality and patient-centeredness of gender-affirming care.^2^ HRQL is assessed through patient-reported outcomes (PROs) captured by validated questionnaires known as PRO measures (PROMs).^3^ PROs provide insights into health care value and symptom changes, addressing potential discrepancies between patient and health care professional perceptions of care and care outcomes.^4,5,6^ Reflecting the importance of PROs, the National Quality Forum in the United States has begun incorporating PROs into quality metrics, and payers and regulators globally are increasingly interested in using PRO data to assess health care value and quality.^7,8,9,10,11,12,13,14^

In gender-affirming care, PROMs have demonstrated utility in measuring improvements in mental health, gender dysphoria, psychosocial outcomes, sexual well-being, and HRQL as well as reductions in anxiety, depression, and suicidality.^15,16,17,18^ For gender-affirming surgery, PROMs have been used to assess postoperative outcomes, including urination, sexual, satisfaction with appearance, and care experience.^19,20,21,22,23,24,25^ However, systematic reviews of PROMs in gender-affirming care have highlighted limitations in the current literature, including reliance on ad hoc measures, use of PROMs validated for cisgender populations, and failure to meet international standards for PROM development.^20,21,26,27,28,29^ The credibility of PROM-based reporting depends on robust psychometric and practical properties within the relevant clinical population. To address gaps in measuring TGD individuals’ outcomes and care experiences, we developed a modular PROM specifically designed for gender-affirming care.

Designing the instrument prioritized creating a PROM grounded in the experiences of individuals seeking gender-affirming care, while ensuring ease of use, comprehensiveness, and international applicability for outcome measurement and benchmarking. The instrument was created using a multistep, mixed-methods approach aligned with established PROM development guidelines.^3,30,31,32,33^ Step 1 involved concept elicitation interviews with adults seeking gender-affirming care from the United States, Canada, Denmark, and the Netherlands, generating a conceptual framework, items, and preliminary scales. Feedback from cognitive debriefing interviews with 7 to 14 patient participants and written or verbal input from 4 to 37 clinicians (number of participants varied by scale) informed iterative refinement. A pilot field test with 601 English-speaking TGD individuals from 30 countries via a crowdsourcing platform called Prolific Academic further refined the scales. Detailed methods and results for step 1 are available elsewhere.^34,35^ To ensure international relevance, the scales were translated into Danish, Dutch, and French-Canadian using best practices for translation and cultural adaptation.^36,37^

This article reports the findings of step 2 of the instrument’s development, an international field test study aimed at identifying the best subset of items to retain in each scale. We also provide an analysis of the psychometric properties of reliability and validity, including construct validity, in TGD adults seeking or receiving gender-affirming care.

## Methods

**Figure 1.**
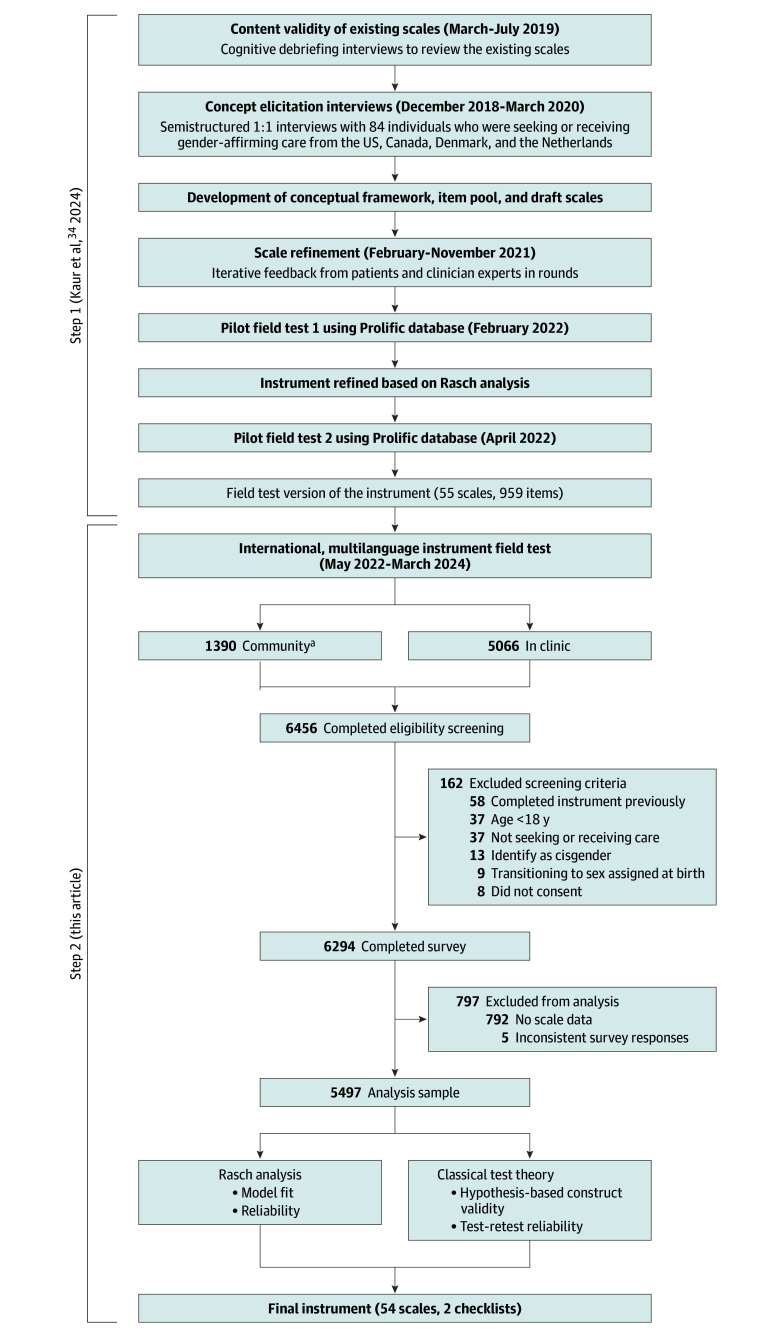
Overview of the Instrument’s Development and Field Test ^a^The community sample included data from pilot field tests 1 and 2.

Ethics approval for the study was obtained from the Hamilton Integrated Research Ethics Board and from collaborating sites, as detailed in eTable 1 in Supplement 1. All participants provided informed consent. The Strengthening the Reporting of Observational Studies in Epidemiology (STROBE) reporting guidelines for cross-sectional studies was followed.^38^ Figure 1 illustrates the recruitment workflow for the study.

### Participants and Procedures

A cross-sectional sample of TGD individuals aged 18 years or older who sought or received gender-affirming care within the past 5 years and could provide informed consent was recruited from 21 clinical sites in Canada, the United States, the Netherlands, and Spain. Participants completed the instrument in English, Danish, Dutch, or French-Canadian (depending on country of recruitment). Recruitment included clinical site–based outreach via emails, social media, patient portals, face-to-face interactions, and posters as well as community-based methods of a crowdsourcing platform (Prolific Academic), outreach to Trans PULSE Canada project participants,^39^ closed TGD-specific social media groups, Trans Pride Australia, Copenhagen Pride, and the developer’s website. Those seeking care for variations in sex characteristics were excluded (eg, Turner syndrome).

Interested participants completed an online questionnaire hosted on REDCap, with data stored on either McMaster University’s or participating sites’ servers, based on each site’s requirements. A 2-step screener confirmed participants were aged 18 years or older, had sought or received gender-affirming care in the past 5 years, and had not previously completed the survey. Eligible participants provided electronic consent. Field test data collection occurred between February 2022 and March 2024, with the pilot field test conducted from February 2022 to April 2022 and the main field test from May 2022 to March 2024. Participants recruited through the Prolific platform were compensated at a prorated rate of $18 per hour. Those recruited through the Trans PULSE Canada research database received a CAD$25 e-gift card. All other participants, upon completing the survey, could choose to enter a draw for 1 of 10 e-gift cards valued at $100 each, unless prohibited by site institutional regulations.

For test-retest (TRT) reliability, participants recruited through Prolific, Trans PULSE Canada, and 3 clinical sites (GrS Montreal, Crane Center for Transgender Surgery, and GU Recon) who consented to be recontacted for future surveys were invited to complete the scales again. At the start of the TRT survey, participants were queried about any changes in health status, appearance, or the construct being measured by the scale since their initial completion. Data from participants who completed the TRT survey between 7 and 14 days after the initial survey and reported no change were included in the analysis. Participants were compensated with a CAD$25 (or equivalent) e-gift card, and those recruited through Prolific were reimbursed at a prorated rate of $18 per hour.

### Measures

Self-reported sociodemographic and clinical data included age, gender identity (open-ended text and check box format), sex assigned at birth, race, education, marital status, sexual orientation, and ability to pay for household expenses and bills. Data on race and ethnicity were collected to describe the sample. Participants indicated the type of gender-affirming care sought or received (to look, function, or feel masculine, feminine, gender fluid, or none of these). Those identifying as gender fluid or none of these were prompted to specify the type of care by area (eg, face, voice, chest, body, genital). Additional questions covered hormone use, voice surgery, voice therapy, and gender-affirming procedures or operations by body part. For each body part, participants indicated past and future surgical plans using options: I am not sure, I do not want this, I want this, I had this, and I had this and need more, along with the duration of time since treatment. Responses for type of care, sex at birth, and gender-affirming procedures and surgeries used skip logic to ensure only relevant scales were completed. Most demographic and clinical questions included a prefer not to answer option, except where responses were essential for skip logic or analysis.

### The PROM Instrument

The field test version of the GENDER-Q comprised of 55 scales and 959 items. Participants completed a core set of scales (n = 12) along with specific scales based on their responses to clinical and sociodemographic variables. Participants had the option to skip items or entire scales if they chose not to respond.

### Statistical Analysis

Sociodemographic and clinical characteristics of the sample were summarized using descriptive statistics (continuous variables as means and SDs; categorical variables as numbers and percentages). Data from the pilot and field test were combined for the psychometric analysis. Rasch Measurement Theory (RMT) analysis, a state-of-the-art modern psychometric approach for developing and refining PROMs, was used to examine the fit of the observed data to the Rasch model for each scale.^40^ RUMM2030 software (RUMM Laboratory) was used with the unrestricted partial credit model for polytomous data. RMT is grounded in a probabilistic framework that models the relationship between a person’s ability or trait level and the difficulty of an item, ensuring that the resulting scales are invariant and interpretable. This approach allows for the construction of unidimensional scales with items that function consistently across groups and provides precise estimates of measurement at both the individual and group level. A series of tests and criteria were applied to determine the optimal subset of items to retain in each scale, aiming to ensure that the scales effectively mapped out a range of measurement for each construct with high reliability and validity (eTable 2 in Supplement 1). Two-sided *P* values of .05 or less were considered significant, with Bonferroni adjustments applied for multiple comparisons where appropriate.

For each scale, items were iteratively examined to determine ordered thresholds, fit to the Rasch model, local dependency, and differential item functioning (DIF). The combined information informed decisions on item retention. For scales with many items exhibiting disordered thresholds, response options were rescored. DIF analyses assessed age groups (18-24, 25-29, 30-39, 40-49, and ≥50 years) and, where applicable, the goal of gender-affirming care (masculinizing or feminizing) to determine whether items functioned consistently across person factors. For scales with more than 500 respondents, the sample size was amended to 500 for tests of fit statistics. To ensure stability, after the final solution for a scale was reached, item fit was reassessed in 5 random samples of 500 participants. To examine reliability, Person Separation Index (PSI) and Cronbach α values were calculated, with reliability values of 0.7 or greater considered sufficient.^41^

Rasch logit scores were transformed to a 0 to 100 scale, where higher scores indicated better outcomes. Classical test theory (CTT) analyses were performed on transformed scores to provide additional evidence of scale performance. Percentage of missing data were computed, based on final item-sets, for each scale. TRT reliability and hypothesis-based construct validity of the scales were examined. For TRT, we aimed to recruit at least 100 participants per scale, the recommended sample size for a very good rating according to COSMIN study design guidelines.^31^ A 2-way mixed-effect model evaluating absolute agreement was used to calculate single and average intraclass correlation coefficient (ICC).^42^ For each scale, ICC values of 0.70 or greater provided evidence of sufficient reliability.^41^ The standard error of measurement and the individual and group level smallest detectable change (SDC) were also calculated.^43^ To assess construct validity, parametric or nonparametric tests were conducted to evaluate the hypothesis that scale scores would increase incrementally with participants reporting better outcomes on the corresponding overall categorical questions for each scale. Categories for the overall questions with fewer than 10 responses in a category were combined for the analysis. The Dilation, Catheter, and Gender Practices scales did not have associated overall questions and were therefore not included in the construct validation analyses. CTT analyses were performed using SPSS version 29 (IBM Corp).

## Results

The sample included 5497 participants with a mean (SD) age of 32.8 (12.3) years (range, 18-83 years). Overall, there were 1837 (33.4%) men, 1307 (23.8%) nonbinary individuals, and 2036 (37.0%) women. Sample characteristics are shown in Table 1. The type of gender-affirming care participants were seeking or had were 2674 (48.6%) masculinization, 2271 (41.3%) femininization, and 552 (10.0%) other. Most participants were from the US or Canada (4191 [76.2%]), never married (3209 [58.4%]), and had completed college-level education or higher (3071 [55.9%]). Overall, 158 participants (2.9%) were Black, 182 (3.3%) Latin American, and 4236 (77.1%) White. When asked about existing mental or physical health conditions diagnosed by a health professional, 3349 participants (60.9%) reported a mental health condition and 1645 (29.9%) reported a physical health condition. Sample treatment characteristics are shown in Table 2. Participants completed a mean (SD) of 22 (8) scales (range, 1-39); mean (SD) scales completed by type of gender-affirming care were: 22 (7) for masculinization; 25 (9), feminization; and 22 (6), other.

**Table 1.** Sociodemographic Characteristics of the Sample

Characteristic	Participants, No. (%) (N = 5497)
Language of the survey	
English	5102 (92.8)
Danish	168 (3.1)
Dutch	118 (2.1)
French Canadian	109 (2.0)
Age at time of survey, y	
18-24	1565 (28.5)
25-29	1230 (22.4)
30-39	1465 (26.7)
40-49	597 (10.9)
≥50	640 (11.6)
Gender identity	
Man	1837 (33.4)
Woman	2036 (37.0)
Nonbinary	1307 (23.8)
Indigenous or other cultural gender minority	49 (0.9)
Another gender	268 (4.9)
Assigned sex at birth	
Male	2363 (43.0)
Female	3134 (57.0)
Goal of the gender-affirming care	
To look, function, or feel more masculine	2673 (48.6)
To look, function, or feel more feminine	2271 (41.3)
To look, function, or feel more gender fluid	481 (8.8)
None of these	69 (1.3)
Missing	3 (0.1)
Intersex	
No	4612 (83.9)
Yes	124 (2.3)
Not sure	704 (12.8)
Prefer not to answer	23 (0.4)
Missing	34 (0.6)
Country of residence	
United States	2440 (44.4)
Canada	1751 (31.9)
United Kingdom	314 (5.7)
Australia	275 (5.0)
Denmark	172 (3.1)
Netherlands	136 (2.5)
Other	402 (7.3)
Prefer not to answer	6 (0.1)
Missing	1 (<0.1)
Race	
Black	158 (2.9)
East Asian	85 (1.5)
Indigenous	43 (0.8)
Latin American	182 (3.3)
Middle Eastern	40 (0.7)
Pacific Islander	6 (0.1)
South Asian	35 (0.6)
Southeast Asian	53 (1.0)
White	4236 (77.1)
Multiple races	561 (10.2)
Unspecified other or unknown	19 (0.3)
Prefer not to answer	61 (1.1)
Missing	18 (0.3)
Difficulty covering household expenses and paying bills in past 3 mo	
Not at all difficult	1841 (33.5)
A little difficult	1335 (24.3)
Somewhat difficult	1135 (20.6)
Very difficult	574 (10.4)
Extremely difficult	384 (7.0)
Missing	228 (4.1)
Marital status	
Never married	3209 (58.4)
Separated	186 (3.4)
Divorced	419 (7.6)
Widowed	32 (0.6)
Living common-law	479 (8.7)
Married	924 (16.8)
Other	187 (3.4)
Prefer not to answer	48 (0.9)
Missing	13 (0.2)
Education level	
Some high school	215 (3.9)
Completed high school	692 (12.6)
Some college or trade school or university	1469 (26.7)
Completed college or trade school or university	2014 (36.6)
Some master’s or doctoral degree	315 (5.7)
Completed master’s or doctoral degree	742 (13.5)
Prefer not to answer	19 (0.3)
Missing	31 (0.6)
Mental health condition diagnosed by clinician that is expected to last or has lasted for at least 6 mo	
No	1886 (34.3)
Yes	3349 (60.9)
Prefer not to answer	229 (4.2)
Missing	33 (0.6)
Physical health condition diagnosed by clinician that is expected to last or has lasted for at least 6 mo	
No	3360 (61.1)
Yes	1645 (29.9)
Prefer not to answer	144 (2.6)
Missing	26 (0.5)
Sexual orientation	
Asexual	724 (13.2)
Bisexual	1646 (29.9)
Gay	668 (12.2)
Lesbian	995 (18.1)
Pansexual	1094 (19.9)
Queer	1788 (32.5)
Questioning or unsure	410 (7.5)
Same-gender loving	289 (5.3)
Straight or heterosexual	929 (16.9)
Other	207 (3.8)
Prefer not to answer	50 (0.9)

**Table 2.** Treatment Characteristics of the Sample

Type of care	Participants, No. (%) (N= 5497)	
	Yes	No
General		
Currently taking hormones	4484 (82.6)	943 (17.4)
Voice		
Voice therapy	1203 (21.9)	4294 (78.1)
Voice surgery	94 (1.7)	5371 (98.3)
Head, face, and neck		
Scalp advancement surgery	139 (3.0)	4556 (97.0)
Surgery or procedure to change shape or size of brow bone	479 (9.6)	4499 (90.4)
Surgery or procedure to change shape or size of nose	415 (8.3)	4559 (91.7)
Surgery or procedure to change shape or size of lips	271 (5.5)	4700 (94.5)
Surgery or procedure to change shape or size of cheeks	172 (3.5)	4807 (96.5)
Surgery or procedure to change shape or size of chin	364 (7.3)	4613 (92.7)
Surgery or procedure to change shape or size of jaws	325 (6.5)	4657 (93.5)
Surgery to reduce Adam’s apple	331 (15.7)	1778 (84.3)
Body		
Chest surgery	2295 (76.2)	718 (23.8)
Breast surgery	574 (25.3)	1698 (74.7)
Surgery to change shape or size of waist	168 (3.1)	5186 (96.9)
Surgery to change shape or size of buttocks	93 (1.7)	5262 (98.3)
Genitals		
Surgery to create a penis	420 (13.7)	2655 (86.3)
Surgery to create a vagina	1334 (58.5)	948 (41.5)

Table 3 presents scale-level results. RMT analysis demonstrated the reliability and validity of 52 of 55 scales measuring aspects of HRQL, sexual, urination, gender practices, voice, hair, face and neck, body, breasts, genital feminization, chest, genital masculinization, and experience of care. The 3 exceptions were binding adverse effects, urinary function, and adverse effects of surgery. For the binding scale, several items exhibited disordered thresholds and did not fit the Rasch model. Removing items with disordered thresholds and splitting the scale into 2 scales—binding skin symptoms and binding chest symptoms—resulted in ordered thresholds and satisfied the requirements of the Rasch model. The surgery adverse effects scale also had multiple items with disordered thresholds. Although collapsing the 2 middle response options yielded acceptable item fit statistics, the data did not fit the Rasch model (*P* < .05), and PSI values were only moderate (≤0.7). For the urinary function scale, based on participants who had undergone genital surgery, nearly all items had disordered thresholds. No satisfactory solution was found that ensured ordered thresholds, good item fit, and acceptable reliability. Consequently, the surgical adverse events and urinary function were deemed checklists.

**Table 3.** Scale-Level RMT Results

Scale	Items, No.	Included in RMT, No./total No. (%)	χ^2^ (*df*)	*P* value	PSI + E	PSI − E	α + E	α − E	DIF	
									Age	M/F
Health-related quality of life										
Body image	8	4102/4525 (90.7)	76.85 (72)	.33	0.96	0.95	0.97	0.96	0	0
Gender dysphoria	14	4014/4519 (88.8)	96.93 (126)	.98	0.94	0.94	0.97	0.96	0	0
Social acceptance	9	3889/4621 (84.2)	77.06 (63)	.11	0.84	0.84	0.92	0.89	0	0
Psychological distress	10	3881/4454 (87.1)	68.29 (90)	.96	0.90	0.91	0.95	0.93	0	0
Psychological well-being	10	4059/4557 (89.1)	68.66 (90)	.95	0.93	0.92	0.95	0.93	0	0
Treatment outcome	10	2516/3469 (72.5)	108.24 (90)	.09	0.84	0.86	0.94	0.91	0	0
Sexual										
Sexual well-being	12	3674/3898 (94.3)	72.53 (108)	>.99	0.92	0.92	0.94	0.93	1; *r* = 1.0	6; *r* = 1.00
Orgasm	8	1313/1470 (89.3)	78.67 (64)	.10	0.90	0.89	0.93	0.91	0	3; *r* = 1.00
Urination										
Urinary catheter	10	199/225 (92.6)	33.54 (20)	.03	0.87	0.84	0.90	0.86	NC	NC
Gender practices										
Binding, well-being	8	326/367 (88.8)	46.97 (40)	.21	0.93	0.91	0.95	0.93	NC	NC
Binding, chest symptoms	10	322/367 (87.7)	52.11 (40)	.10	0.87	0.87	0.93	0.91	NC	NC
Binding, skin symptoms	5	303/366 (82.8)	39.88 (20)	.01	0.74	0.73	0.85	0.80	NC	NC
Tucking, symptoms	10	239/306 (78.1)	47.42 (30)	.02	0.78	0.80	0.91	0.87	NC	NC
Voice										
Sound	15	5129/5415 (94.7)	85.83 (135)	>.99	0.96	0.96	0.97	0.96	0	7
Distress	10	4794/5367 (89.3)	83.87 (90)	.66	0.92	0.93	0.95	0.95	0	0
Hair										
Hair-face F	7	1315/1584 (83)	84.81 (63)	.04	0.90	0.90	0.95	0.93	0	NA
Hair-face M	12	1887/2043 (92.4)	133.21 (108)	.05	0.95	0.95	0.96	0.96	0	NA
Hair-head	12	1592/1703 (93.5)	108.7 (108)	.46	0.94	0.94	0.96	0.95	0	8
Face and Neck										
Face overall	15	4557/4898 (93.0)	97.89 (135)	.99	0.97	0.97	0.98	0.97	0	0
Facial features	9	4466/4854 (92.0)	61.28 (81)	.95	0.91	0.90	0.93	0.90	0	6
Upper face	9	1441/1582 (91.1)	60.37 (81)	.96	0.96	0.96	0.97	0.96	0	2; *r* = 1.00
Eyebrows	5	1402/1564 (89.6)	37.07 (40)	.60	0.90	0.88	0.93	0.89	0	1
Cheeks	9	773/866 (89.3)	46.12 (54)	.77	0.97	0.96	0.98	0.97	0	0
Nose	10	1293/1438 (89.9)	110.57 (90)	.07	0.96	0.96	0.98	0.96	0	0
Nostrils	7	1164/1355 (85.9)	35.32 (49)	.93	0.96	0.95	0.98	0.96	0	0
Lips	12	975/1050 (92.9)	144.24 (108)	.01	0.97	0.96	0.98	0.97	0	2; *r* = 1.00
Chin	10	1313/1496 (87.8)	111.4 (90)	.06	0.97	0.97	0.99	0.97	0	1; *r* = 1.00
Jawline	10	1698/1905 (89.1)	109.3 (90)	.08	0.97	0.96	0.98	0.97	0	3; *r* = 1.00
Adam’s apple	10	810/1146 (70.7)	72.13 (80)	.72	0.94	0.95	0.98	0.96	0	NA
Body										
Body	10	4709/4973 (94.7)	30.8 (90)	>.99	0.95	0.95	0.96	0.96	0	2; *r* = 1.00
Buttocks	10	1260/1406 (89.6)	72.3 (90)	.91	0.96	0.96	0.98	0.97	0	1; *r* = 1.00
Waist	7	1948/2225 (87.6)	43.78 (56)	.88	0.94	0.93	0.96	0.94	0	2; *r* = 1.00
Breasts										
Breasts	12	2041/2131 (95.8)	107.62 (108)	.49	0.93	0.93	0.95	0.94	0	NA
Nipples and areola	8	1777/2071 (85.5)	109.7 (72)	.003	0.91	0.89	0.94	0.91	0	NA
Genital F										
Vagina	10	1106/1236 (89.5)	118.03 (90)	.03	0.89	0.87	0.92	0.89	0	NA
Labia	12	1027/1152 (89.1)	119.67 (108)	.21	0.94	0.93	0.96	0.94	0	NA
Clitoris	6	925/1118 (82.7)	38.69 (36)	.35	0.92	0.90	0.95	0.92	0	NA
Dilation	5	836/930 (89.9)	27.08 (35)	.83	0.89	0.87	0.92	0.88	0	NA
Chest										
Chest	10	2186/2857 (76.5)	95.45 (90)	.33	0.94	0.95	0.99	0.98	0	NA
Scars	12	1340/1927 (69.5)	113.51 (108)	.34	0.81	0.85	0.95	0.92	0	NA
Nipples and areola	8	2200/2535 (86.9)	71.4 (72)	.50	0.92	0.91	0.95	0.93	0	NA
Genital M										
Penis	12	366/391 (93.6)	98.74 (60)	.001	0.93	0.93	0.96	0.95	NC	NA
Penis sensation	11	250/281 (89.0)	36.14 (33)	.32	0.95	0.95	0.97	0.95	NC	NA
Glans	9	194/223 (87.0)	19.56 (18)	.36	0.93	0.92	0.96	0.94	NC	NA
Scrotum	10	287/310 (92.6)	70.87 (40)	.002	0.93	0.93	0.95	0.94	NC	NA
Perineum	8	133/174 (76.4)	19.64 (16)	.24	0.90	0.89	0.96	0.92	NC	NA
Donor site, forearm or thigh	8	214/252 (84.9)	34.25 (24)	.08	0.90	0.89	0.95	0.92	NC	NA
Donor site, adverse effects	12	190/251 (75.7)	43.01 (24)	.009	0.70	0.73	0.88	0.84	NC	NA
Testicular implants	10	79/95 (83.2)	12.67 (20)	.89	0.88	0.83	0.93	0.88	NC	NA
Erectile device	12	77/78 (98.7)	22.42 (24)	.55	0.90	0.90	0.93	0.93	NC	NA
Experience of care										
Health professional	15	1308/3017 (43.4)	99.87 (90)	.22	0.74	0.90	0.98	0.97	0	0
Clinic	10	1022/2333 (43.8)	100.71 (70)	.009	0.74	0.88	0.97	0.94	0	0
Surgery, information	10	364/530 (68.7)	57.23 (40)	.04	0.84	0.88	0.95	0.92	NC	NC
Surgery, return to activity	12	280/594 (47.1)	55.4 (48)	.22	0.79	0.91	0.97	0.95	NC	NC

Abbreviations: DIF, differential item functioning; E, extremes; F, feminization; M, masculinization; NA, not applicable; NC, not calculated due to sample size <300; PSI, Person Separation Index; RMT, Rasch Measurement Theory.

RMT analysis reduced the number of items in the 54 scales by 60.5%, from 904 to 547 items. Items in the treatment outcome, urinary catheter, tucking symptoms, chest scar, vagina, testicular implants, perineum, erectile device, and donor-site adverse events were rescored, resulting in ordered thresholds for all items in the final versions of the scales. For each scale, all items in their final version fit the Rasch model with nonsignificant χ^2^ *P* values after Bonferroni adjustment. Item fit residuals greater than 0.30 were observed for 183 item pairs across 51 scales, indicating some local dependency; however, subtest analyses showed marginal impact on scale reliability (≤0.13 difference in PSI value). None of the PSI values (with and without extremes) dropped below 0.70, except for the clinic scale, where the PSI values with extremes fell to 0.63. For all scales, item coverage was adequate, with no substantial gaps in the measured construct and limited clustering of items. For the 48 scales analyzed for DIF by age group, DIF was detected in 4 items; for the 28 scales analyzed by treatment goal, 47 items showed DIF. When items were split based on DIF variables, Pearson correlations between original and the new split person locations indicated DIF had a negligible impact (all Pearson correlations ≥0.95). For the 35 scales completed by more than 500 participants, 5 random samples of 500 participants provided broad support for the final version of the scales.

The scales demonstrated good targeting as the percentage of sample who scored on the outcome scales was high (HRQL, ≥72.5%; sexual, ≥89.3%; urination, ≥92.6%; gender practices, ≥78.1%; voice, ≥89.3%; hair, ≥83.0%; face and neck, ≥70.7; body, ≥87.6%; breast, ≥85.5%; genital feminization, ≥82.7%; chest, ≥69.5%; and genital masculinization, ≥76.4%). The PSI with extremes and without extremes was high (>0.85) for 45 scales and for 49 scales, respectively. For the remaining scales, the PSI was moderate (0.70-0.85), and internal consistency was excellent with Cronbach α values (with and without extremes) of 0.80 or greater.

The CTT analyses showed strong evidence of instrument’s reliability and hypothesis-based construct validity. Detailed results for TRT are provided in eTable 3 in Supplement 1 for both single and average ICCs. For TRT, the sample size met COSMIN criteria for a very good rating (≥100) for 34 scales, adequate (50-99) for 1 scale, doubtful (30-49) for 8 scales, and inadequate (<30) for 8 scales. All scales except the phalloplasty donor flap scale (ICC, 0.65; 95% CI, 0.14-0.86) had an ICC (average) of greater than 0.70, ranging from 0.73 (95% CI, 0.30-0.89) for genital masculinization, donor site adverse effects to 0.98 (95% CI, 0.96-0.99) for genital masculinization, penis sensation. Group SDC ranged from 1.0 to 8.4. The catheter and surgery information scales were excluded from the TRT analysis due to the limited number of participants completing these scales (<5). For construct validity, scale scores increased incrementally with better self-reported responses to the overall construct questions. For example, among 661 participants who reported poor psychological well-being, the mean (SD) scale score was 45 (18) points; for those who reported excellent psychological well-being, the mean (SD) scale score was 85 (16) points (*P* < .001) (eTable 4 in Supplement 1). eTables 5 and 6 in Supplement 1 show the proportion of participants to report problems on the items on the urinary function and adverse effects checklist, respectively. eTable 7 in Supplement 1 provides the sample that was used for the analysis for each scale, and the mean scores and key demographic characteristics for the scales.

## Discussion

**Figure 2.**
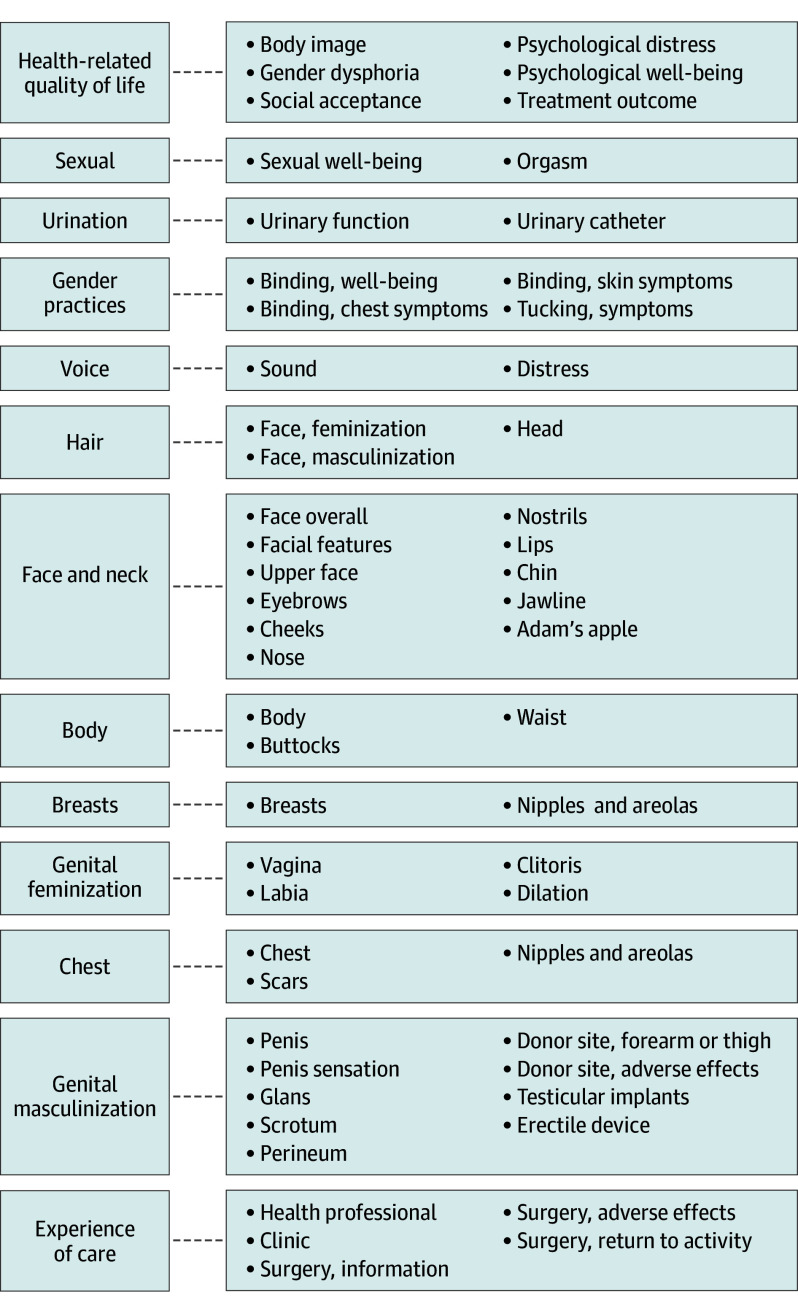
Conceptual Framework of the Patient-Reported Outcome Measure Instrument

This instrument is a rigorously developed, modular PROM designed for individuals 18 years and older seeking gender-affirming care, adhering to established PROM development guidelines. It comprises 54 unidimensional scales and 2 checklists, covering a comprehensive range of PROs (Figure 2) relevant to gender-affirming care. A mixed-methods approach grounded in extensive input from an international sample of TGD individuals and gender-affirming care clinicians ensured content validity, while psychometric validation confirmed the instrument’s reliability and validity.

The instrument addresses the need for a validated, gender-affirming PRO assessment tool for TGD individuals.^20,21,27,28,29^ Unlike existing measures that are developed ad hoc or adapted from cisgender populations, this PROM was developed with TGD individual’s input at every stage, including concept elicitation, scale refinement, and international field testing. The use of RMT offers distinct advantages over the commonly used CTT in TGD PROMs, enabling interval-level scoring, improved item function analysis, and generalizability.^44,45^ RMT and CTT together robustly established the instrument’s reliability and validity for use in gender-affirming care.

To our knowledge, this is the only gender-affirming care–specific PROM developed with a large international sample of TGD individuals. Its modular design allows users to select relevant scales, facilitating integration into clinical care and research while minimizing patient and clinician burden. As the field evolves, new scales can be added to address emerging needs.

The literature provides examples of how PROMs have been used in health care and social care.^46,47,48,49^ The PROM described in this article may be used to support clinical care by aiding in screening, risk stratification, expectation management, goal setting, monitoring health status, and facilitating communication between patients and clinicians. Aggregated data could be used to inform care delivery, evaluate interventions, and support health policy decisions, promoting value-based gender-affirming care.

### Limitations

The limitations of the field test’s design and sample must be considered. The field test relied on self-reported data and online data collection via REDCap, which may have excluded individuals with limited technology skills or access or those living in unsafe or unsupportive environments. Additionally, those unable to engage with lengthy surveys due to time constraints or fatigue may have been excluded, despite the option to save and return later. While the study benefits from an international sample of TGD adults, the predominantly White sample limits generalizability to racially, ethnically, and geographically diverse populations. This demographic pattern reflects broader challenges in diversifying research samples in gender-affirming care, where structural inequities may limit access to both care and research participation. This limitation is particularly relevant as experiences and outcomes of TGD individuals may vary across sociocultural and systemic contexts.

Additionally, small sample sizes constrained testing for some scales. The proportion of participants scoring on the experience of care scales was low (43.4%-68.7%), consistent with literature indicating high ceiling effects in health care experience evaluations.^50,51,52,53^ For TRT, the sample size met COSMIN criteria for a very good rating (≥100) for 34 scales, adequate for 1 scale, doubtful for 8 scales, and inadequate for 8 scales. Furthermore, the TRT for urinary catheter and information scales were not tested due to small sample size. Although the instrument was validated in 4 languages, establishing the cross-cultural validity of the PROM will be critical to ensuring its robustness and utility in diverse global contexts. Future publications will further examine the construct validity of the scales, while additional studies could assess their reliability in independent clinical samples.

## Conclusions

This novel PROM instrument consisting of 54 independently functioning scales and 2 checklists demonstrated reliability and validity in a large international sample of TGD adults. This is a rigorously developed instrument for use in gender-affirming care, research, quality improvement, and regulatory efforts and is available online.^54^

## Notes

Supplement 1. **eTable 1.** Field Test Sites **eTable 2.** RMT Criteria and Statistical Tests **eTable 3. **Test-Retest Reliability Results **eTable 4.** Construct Validity **eTable 5.** Distribution of Urinary Function Checklist **eTable 6.** Distribution of Surgery, Adverse Effect Checklist **eTable 7.** Mean Scores and Key Demographic Characteristics for Scales

Supplement 2.
Data Sharing Statement
