# Supplementary material for: Development and Assessment of a Patient-Reported Outcome Instrument for Gender-Affirming Care
Source: JAMA Netw Open. 2025 Apr 18;8(4):e254708. doi: 10.1001/jamanetworkopen.2025.4708 (PMC12008761; doi:10.1001/jamanetworkopen.2025.4708)
Supplement: Supplement 1. — eTable 1. Field Test Sites eTable 2. RMT Criteria and Statistical Tests eTable 3. Test-Retest Reliability Results eTable 4. Construct Validity eTable 5. Distribution of Urinary Function Checklist eTable 6. Distribution of Surgery, Adverse Effect Checklist eTable 7. Mean Scores and Key Demographic Characteristics for Scales [file jamanetwopen-e254708-s001.pdf]

Supplemental Online Content

Kaur MN, Rae C, Morrison SD, et al. Development and assessment of a patient-reported outcome instrument for gender-affirming care. *JAMA Netw Open.* 2025;8(4):e254708. doi:10.1001/jamanetworkopen.2025.4708

eTable 1. Field Test Sites

eTable 2. RMT Criteria and Statistical Tests

eTable 3. Test-Retest Reliability Results

eTable 4. Construct Validity

eTable 5. Distribution of Urinary Function Checklist

eTable 6. Distribution of Surgery, Adverse Effect Checklist

eTable 7. Mean Scores and Key Demographic Characteristics for Scales

This supplemental material has been provided by the authors to give readers additional information about their work.

© 2025 Kaur MN et al. *JAMA Network Open*.

eTable 1. Field Test Sites

	Organization name/location	Survey distribution method	REDCap survey hosted at McMaster University
Crowdsourcing / existing research databases	Prolific	Participants accessed survey through Prolific platform	Yes
	Transpulse Survey Canada, Western University, London, Ontario	Emailed survey link to database participants	Yes
Social Media Recruitment Only	Flinders University, Adelaide, South Wales, Australia	Shared survey link through X (Twitter), Facebook, Reddit, Discord platforms for LGBTQ2+ community support groups	Yes
	Odense University Hospital, Research Unit for Plastic Surgery, Odense, Denmark	Shared survey link at Copenhagen PRIDE event<br>Shared survey link through Facebook groups for LGBTQ2+ community support groups	Danish survey – No<br>English survey – Yes
	East of England Gender Service, Cambridge, United Kingdom	Shared survey link through LGBTQ2+ community support groups (e.g., OUTpatients)	Yes
Clinical Sites			
Canada	GrS Montreal, Montreal, Quebec	Emailed survey link to retrospective patient list	Yes
	McLean Clinic, Mississauga, Ontario	Emailed survey link to retrospective patient list	Yes
	Women's College Hospital, Trans-related Surgery Center, Toronto, Ontario	Emailed survey link to retrospective patient list	Yes
United States	Align Surgical Associates, San Francisco, California	Emailed survey link to retrospective patient list	Yes
	Boston Children's Hospital, Center for Gender Surgery, Boston, Massachusetts	Emailed survey link to retrospective patient list	No
	Brigham and Women's Hospital, Harvard Medical School, Boston, Massachusetts	Emailed survey link to retrospective patient list	No
	Cedars-Sinai Medical Center, Department of Surgery, Los Angeles, California	Emailed survey link to retrospective patient list	Yes
	Crane Center for Transgender Surgery, Austin, Texas	Emailed survey link to retrospective patient list	Yes
	G.U. Recon Clinic, San Francisco, California	Emailed survey link to retrospective patient list	Yes
	Johns Hopkins, Baltimore Maryland	Emailed survey link to retrospective patient list	Yes
	MedStar Georgetown University Hospital, Washington D.C.	Emailed survey link to retrospective patient list<br>In-person recruitment in clinic	Yes
	New York University Langone Health, New York, New York	Emailed survey link to retrospective patient list	No
	Oregon Health and Sciences University, Portland, Oregon	Emailed survey link to retrospective patient list	Yes

© 2025 Kaur MN et al. *JAMA Network Open*.

	Rebirth Health Center, Salt Lake City, Utah	Advertising flyers in clinic<br>Social media – posted survey link on clinic's Facebook page	Yes
	Rush University Medical Center, Rush Gender Affirmation Surgery, Chicago, Illinois	Advertising flyers in clinic	Yes
	Temple University, Lewis Katz School of Medicine, Philadelphia, Pennsylvania	Emailed survey link to retrospective patient list	Yes
	University of Colorado, UCHealth Plastic and Reconstructive Surgery Clinic – Anschutz Medical Campus, Aurora, Colorado	Emailed survey link to retrospective patients<br>In-person recruitment in clinic or by telephone with current patients	No
	University of Michigan, Department of Plastic Surgery, Ann Arbor, Michigan	Emailed survey link to retrospective patient list	No
	Yale University, Plastic & Reconstructive Surgery, New Haven, Connecticut	In-person recruitment in clinic	No
The Netherlands	Amsterdam University Medical Center, Center for Expertise on Gender Dysphoria, Amsterdam	Emailed survey link to retrospective patient list<br>In-person recruitment in clinic	No
Spain	FacialTeam Group, HC Marbella International Hospital, Malaga	Emailed survey link to retrospective patient list<br>Social media – posted survey link on clinic's X (Twitter), Instagram, LinkedIn and Facebook pages<br>Survey link shared during Live interview with GENDER-Q Study Investigators on FacialTeam members-only Youtube Channel	Yes

© 2025 Kaur MN et al. *JAMA Network Open*.

eTable 2. RMT Criteria and Statistical Tests

**Response threshold order** – evaluates if the response categories are ordered such that as the latent trait (ability) increases, the probability of endorsing a response category aligned with more of the latent trait also increases in a predictable manner, i.e., more individuals should endorse a lower level of the latent trait and fewer should endorse a higher level of the latent trait. The number of thresholds is equal to the number of response options minus one. Disordered thresholds may indicate that there are too many response options, or that the labels for response options are confusing.
**Item fit** – evaluates the extent to which the observed data fit the expectations of the Rasch model. When data fit the model, the items should have a hierarchical order such that items that indicate lower levels of latent trait are at the lower end of the Rasch "ruler" and items that indicate higher levels of the latent trait are at the higher end. Item fit is indicated by non-significant Chi-square after Bonferroni adjustment and fit residuals that are ideally within the range -2.5 and +2.5. Item fit is also examined graphically using item characteristic curves that show the probability of a correct response across different levels of the latent trait. The goal is to have well-fitting items with smooth, monotonically increasing item characteristic curves.
**Local dependency –** indicates the extent to which the response to an item influences the response to another item in an item set. The inter-relatedness of items, if detected, impacts the probabilistic structure of the Rasch model and inflates the other psychometric properties of the scale (i.e., reliability and validity). Items with pair-wise residual correlations higher than 0.3 indicate local dependence. Locally dependent items are evaluated in a subtest to determine their impact on scale's reliability.
**Targeting –** evaluates the spread of person locations and item locations. Person locations for a well targeted scale are centred at zero and have a standard deviation of 1. This is inspected graphically with person-item threshold plots with the goal to have limited clustering of items and gaps on the scale. A scale that is well targeted has more coverage and has the mean person location close to the center of the items. The proportion of a sample that scores on the scale's range of measurement can also be determined.

© 2025 Kaur MN et al. *JAMA Network Open*.

**Differential item functioning (DIF) –** evaluates if the item difficulty hierarchy is consistent across subgroups of people being measured. DIF is assessed with a significant F-test from a two-way analysis of variance and graphically using item characteristic curves. For GENDER-Q, DIF was examined for the following characteristics: age (18-24, 25-29, 30-39, 40-49, 50 years) and the goal of gender-affirming care (masculinizing or feminizing). DIF was computed for scales after selecting a random sample of equal sized subgroups. DIF was performed as long as the subgroups included at least 50 participants, and the overall sample size was at least 300. Items that evidenced significant was split on the sample characteristic. Person correlations between the original and the new person locations were computed to examine the impact of DIF on scoring.
**Reliability -** indicates how well a scale can distinguish between different levels of person's ability. This form of reliability is assessed with the person separation index (PSI), where higher values indicate better discrimination. A scale with higher PSI values facilitates measurement of change. Values >0.9 are considered suitable for measuring within-person change and values >0.7 are suitable for detecting group differences. Cronbach alpha is used to examine internal reliability. For test-retest reliability, intraclass correlation coefficients (ICC) with a two-way random effects model are used to evaluate the consistency of responses 7–14 days after the initial scale completion, barring changes in status for the construct measured by the scale.
**Hypothesis-based construct validity** - refers to the extent to which the scale accurately measures the construct it purports to measure. Parametric or nonparametric tests were used depending on the distribution of the data. Rasch transformed scale scores (0-worse, 100-best) were used. It is of major importance that the hypotheses are defined in advance when assessing construct validity to enable one to draw unbiased conclusions after data collection.

© 2025 Kaur MN et al. *JAMA Network Open*.

eTable 3. Test-Retest Reliability Results

Scale	N	Valid n	ICC Single	95% Confidence interval		ICC Average	95% Confidence interval		Means						SEM	SDC individual	SDC group
				LB	UB		LB	UB	T1	T1 SD	T2	T2 SD	Mean diff	Mean diff SD	$Sd_{pooled}*\sqrt{(1 - ICC)}$	$1.96*\sqrt{2}*SEM$	$SDC_{ind}/\sqrt{n}$
HEALTH-RELATED QUALITY OF LIFE																	
Body Image	125	106	0.77	0.68	0.84	0.87	0.81	0.91	43	21	44	21	1.8	14.2	7.6	21.0	2.0
Gender Dysphoria	125	87	0.84	0.76	0.89	0.91	0.86	0.94	53	17	56	18	2.6	9.6	5.2	14.3	1.5
Social Acceptance	125	93	0.79	0.70	0.86	0.88	0.82	0.92	64	12	64	13	0.0	8.0	4.2	11.7	1.2
Psychological Distress	125	87	0.74	0.63	0.82	0.85	0.77	0.90	55	19	56	20	0.9	14.2	7.6	21.0	2.3
Psychological Well-Being	125	88	0.73	0.62	0.82	0.85	0.77	0.90	50	17	51	17	1.5	12.4	6.7	18.6	2.0
Treatment Outcome	144	123	0.81	0.73	0.86	0.89	0.85	0.93	71	21	68	22	-3.2	13.0	7.0	19.3	1.7
SEXUAL																	
Sexual Well-Being	125	106	0.79	0.71	0.85	0.88	0.83	0.92	55	17	55	14	-0.2	10.2	5.4	15.0	1.5
Orgasm	125	106	0.85	0.78	0.90	0.92	0.87	0.95	61	17	64	18	2.8	9.1	5.0	13.8	1.3
GENDER PRACTICES																	
Binding Well-Being	111	107	0.74	0.60	0.83	0.85	0.75	0.91	66	17	61	18	-5.1	12.0	6.8	18.9	1.8
Binding Adverse - Body	110	100	0.77	0.67	0.84	0.87	0.80	0.91	68	16	65	16	-2.8	10.3	5.7	15.8	1.6
Binding Adverse - Skin	110	100	0.71	0.60	0.80	0.83	0.75	0.89	73	20	72	18	-1.9	14.5	7.9	21.8	2.2
Tucking Adverse	36	29	0.76	0.56	0.88	0.87	0.72	0.94	75	19	73	17	-1.8	12.5	6.6	18.3	3.4
VOICE																	
Sound	125	111	0.80	0.71	0.86	0.89	0.83	0.92	48	14	50	18	2.1	10.2	5.4	15.0	1.4
Distress	125	111	0.79	0.71	0.85	0.89	0.83	0.92	57	19	59	21	2.0	13.1	6.9	19.0	1.8
HAIR																	
Face- Feminization	110	28	0.91	0.82	0.96	0.95	0.90	0.98	42	16	43	15	0.8	6.6	3.4	9.3	1.8
Face- Masculinization	108	47	0.76	0.61	0.86	0.86	0.76	0.92	50	18	51	21	1.4	13.6	7.2	19.9	2.9
Head	124	105	0.85	0.77	0.90	0.92	0.87	0.94	62	20	59	19	-3.0	10.6	5.7	15.8	1.5
FACE & NECK																	
Face Overall	124	105	0.82	0.75	0.88	0.90	0.86	0.93	47	14	48	16	1.1	9.1	4.7	13.1	1.3
Facial Features	124	105	0.79	0.71	0.85	0.88	0.83	0.92	53	13	54	13	1.3	8.5	4.4	12.3	1.2
Upper Face	124	105	0.77	0.68	0.84	0.87	0.81	0.91	57	18	56	19	-1.0	12.3	6.6	18.3	1.8
Eyebrows	124	105	0.83	0.76	0.88	0.91	0.86	0.94	61	20	63	18	1.9	11.1	5.9	16.2	1.6

© 2025 Kaur MN et al. *JAMA Network Open*.

Cheeks	124	105	0.82	0.74	0.87	0.90	0.85	0.93	54	20	54	21	0.5	12.3	6.4	17.8	1.7
Nose	124	105	0.83	0.76	0.88	0.91	0.87	0.94	57	21	58	21	0.3	12.1	6.3	17.4	1.7
Nostrils	124	105	0.74	0.64	0.82	0.85	0.78	0.90	63	20	63	21	-0.1	14.9	7.9	21.8	2.1
Lips	124	105	0.79	0.71	0.86	0.89	0.83	0.92	59	19	61	20	1.7	12.3	6.6	18.2	1.8
Chin	124	105	0.75	0.66	0.83	0.86	0.79	0.91	53	21	52	22	-0.9	15.2	8.1	22.4	2.2
Jawline	124	105	0.85	0.78	0.89	0.92	0.88	0.94	47	22	49	22	1.7	12.2	6.3	17.6	1.7
Adam's Apple	97	83	0.90	0.85	0.93	0.95	0.92	0.97	64	27	64	26	-0.6	12.1	6.1	17.0	1.9
BODY																	
Body	125	106	0.81	0.73	0.87	0.89	0.84	0.93	38	17	40	18	2.3	10.5	5.7	15.7	1.5
Buttocks	124	105	0.88	0.82	0.92	0.94	0.90	0.96	46	22	50	23	3.1	10.8	5.7	15.8	1.5
Waist	124	105	0.75	0.65	0.82	0.86	0.79	0.90	38	23	42	21	3.1	15.4	8.4	23.2	2.3
BREAST																	
Breast	133	107	0.86	0.80	0.90	0.92	0.89	0.95	50	19	52	19	1.6	9.9	5.2	14.4	1.4
Nipples & Areolas	132	107	0.85	0.79	0.90	0.92	0.88	0.94	61	20	61	19	-0.3	10.8	5.5	15.4	1.5
GENITAL FEMINIZATION																	
Vagina	39	27	0.89	0.77	0.95	0.94	0.87	0.97	57	18	58	22	0.6	9.6	4.9	13.5	2.6
Labia	38	26	0.89	0.77	0.95	0.94	0.87	0.97	56	15	58	15	1.8	7.0	3.7	10.3	2.0
Clitoris	37	25	0.87	0.74	0.94	0.93	0.85	0.97	63	21	65	25	1.4	11.9	6.0	16.7	3.3
Dilation	33	21	0.69	0.37	0.86	0.82	0.54	0.93	63	16	62	14	-1.0	12.3	6.5	18.1	4.0
CHEST																	
Chest	226	208	0.93	0.91	0.95	0.96	0.95	0.97	49	31	49	27	0.2	11.0	5.6	15.6	1.1
Nipples & Areolas	226	209	0.88	0.84	0.90	0.93	0.91	0.95	53	21	55	21	1.3	10.3	5.4	15.0	1.0
Scars	112	97	0.90	0.86	0.94	0.95	0.93	0.97	80	18	79	17	-0.9	7.7	3.9	10.8	1.1
GENTIAL MASCULINIZATION																	
Penis	42	30	0.90	0.79	0.95	0.95	0.89	0.97	62	15	63	16	0.6	7.1	3.6	10.0	1.8
Penis Sensation	41	34	0.96	0.93	0.98	0.98	0.96	0.99	60	28	63	26	2.1	7.2	3.7	10.3	1.8
Glans	26	18	0.89	0.72	0.96	0.94	0.83	0.98	72	21	76	20	4.0	9.2	5.0	13.8	3.3
Scrotum	34	25	0.91	0.81	0.96	0.95	0.90	0.98	66	19	66	16	0.6	7.5	3.8	10.6	2.1
Perineum	23	19	0.82	0.59	0.93	0.90	0.75	0.96	69	24	72	25	3.4	14.9	7.8	21.7	5.0
Donor Site - Forearm or Thigh	28	21	0.48	0.08	0.75	0.65	0.14	0.86	66	25	71	21	5.0	24.1	13.8	38.3	8.4
Donor Site - Adverse Effects	27	20	0.57	0.18	0.81	0.73	0.30	0.89	80	16	80	14	0.8	14.0	7.8	21.7	4.9
Testicular Implants	12	11	0.88	0.62	0.97	0.94	0.76	0.98	62	16	62	19	0.2	8.8	4.4	12.2	3.7
Erectile Device	10	6	0.90	0.50	0.99	0.95	0.67	0.99	60	15	57	16	-2.7	7.3	3.7	10.1	4.1

© 2025 Kaur MN et al. *JAMA Network Open*.

EXPERIENCE CARE																
Health Professional	152	80	0.72	0.59	0.81	0.84	0.75	0.90	78	22	76	22	-2.3	16.5	8.9	24.6	2.8
Clinic	105	58	0.78	0.65	0.86	0.87	0.79	0.93	79	21	78	19	-1.2	13.4	7.1	19.7	2.6
Surgery - Return to Activity	11	9	0.57	-0.02	0.88	0.72	-0.04	0.94	88	16	96	10	7.9	11.4	7.1	19.6	6.5

ICC, Intraclass correlation co-efficient; UB, upper bound; LB, lower bound; T1, time 1; T2, time 2 (7-14 days after T1); SEM, standard error of measurement; SDC, smallest detectable change; SD, standard deviation

© 2025 Kaur MN et al. *JAMA Network Open*.

eTable 4. Construct Validity

eTable 4a. RESULTS FOR HYPOTHESIS-BASED CONSTRUCT VALIDITY TESTING OF THE GENDER-Q SCALES

Scale	Response	N	Mean	Standard deviation	Standard error	95% CI		p-value
						LB	UB	
In general, how would you rate your satisfaction with your social activities and relationships?								
Gender Dysphoria	Poor	628	49	19	1	48	51	<0.001
	Fair	1081	58	18	1	57	59	
	Good	1394	63	19	1	62	64	
	Very Good	967	69	19	1	68	70	
	Excellent	384	78	21	1	76	80	
Social Acceptance	Poor	647	60	17	1	59	61	<0.001
	Fair	1107	68	15	0	67	69	
	Good	1428	74	16	0	74	75	
	Very Good	988	80	15	0	79	81	
	Excellent	397	88	13	1	87	89	
In general, how would you rate your mental health, including your mood and your ability to think?								
Psychological Distress	Poor	644	48	21	1	46	50	<0.001
	Fair	1211	59	19	1	58	60	
	Good	1291	67	18	1	66	68	
	Very Good	905	74	17	1	72	75	
	Excellent	381	83	17	1	81	85	
Psychological Well-Being	Poor	661	45	18	1	43	46	<0.001
	Fair	1233	55	16	0	54	56	
	Good	1324	64	16	0	63	65	
	Very Good	927	72	16	1	71	73	
	Excellent	386	85	16	1	83	86	
How satisfied are you with your sex life?								
Sexual Well-Being	Not at all satisfied	416	45	14	1	43	46	<0.001
	A little satisfied	705	51	12	0	50	52	

© 2025 Kaur MN et al. *JAMA Network Open*.

	Somewhat satisfied	1293	58	12	0	57	58	
	Very satisfied	952	67	15	0	66	68	
	Extremely satisfied	462	77	17	1	75	78	
When you engage in sexual activity (e.g., masturbation, partnered sex), are you able to have an orgasm?								
Orgasm	Rarely	224	37	16	1	35	39	<0.001
	Sometimes	666	50	13	1	49	51	
	Often	1432	61	12	0	61	62	
	Always	1381	75	17	0	74	76	
Your donor area overall?								
Donor Site - Forearm or Thigh	Not at all bothered	74	88	13	1	85	91	<0.001
	A little bothered	91	66	10	1	64	68	
	Somewhat bothered	42	53	7	1	51	56	
	Very bothered	27	44	10	2	40	48	
	Extremely bothered	17	21	23	6	9	33	
Your donor area scar overall?								
Donor Site - Adverse Effects	Not at all concerned	149	88	11	1	86	90	<0.001
	A little concerned	67	67	10	1	64	69	
	Somewhat concerned	22	62	17	4	55	69	
	Very concerned	7	63	18	7	46	80	
	Extremely concerned	6	33	20	8	13	54	
How much feeling do you have in your penis?								
Penis Sensation	I have no feeling	30	11	14	3	6	16	<0.001
	I have a little feeling	70	32	11	1	30	35	
	I have some feeling	97	45	10	1	43	47	
	I have a lot of feeling	97	62	13	1	59	64	
	I have complete feeling	87	90	15	2	86	93	
Your chest scars overall?								
Chest - Scars	Not at all bothered	1080	91	11	0	90	92	<0.001
	A little bit bothered	668	68	10	0	68	69	
	Quite a bit bothered	118	56	11	1	54	58	

© 2025 Kaur MN et al. *JAMA Network Open*.

	Very much bothered	54	36	21	3	30	41	
How your Adam's apple looks overall?								
Adam's Apple	Not at all bothered	1031	98	5	0	98	99	<0.001
	A little bothered	456	72	11	0	71	73	
	Somewhat bothered	238	53	10	1	52	55	
	Very bothered	131	41	14	1	39	43	
	Extremely bothered	94	19	18	2	15	22	
Overall, I am completely satisfied with the care I received from my health professional.								
Health Professional	Strongly disagree	64	29	17	2	25	34	p<0.001
	Mostly disagree	55	44	9	1	41	46	
	Slightly disagree	71	54	8	1	52	56	
	Slightly agree	140	60	11	1	59	62	
	Mostly agree	584	75	13	1	74	76	
	Strongly agree	2086	97	7	0	96	97	
Overall, I am completely satisfied with the clinic.								
Clinic	Strongly disagree	33	34	23	4	25	42	p<0.001
	Mostly disagree	45	49	14	2	45	53	
	Slightly disagree	65	55	13	2	52	58	
	Slightly agree	164	63	13	1	61	65	
	Mostly agree	510	76	14	1	75	78	
	Strongly agree	1506	97	7	0	97	97	
Overall, I am completely satisfied with the information I received about my gender-affirming surgery.								
Surgery - Information	Not at all satisfied	14	36	12	3	29	44	p<0.001
	A little satisfied	32	47	7	1	44	49	
	Somewhat satisfied	81	55	9	1	53	57	
	Very satisfied	165	68	11	1	66	70	
	Extremely satisfied	236	94	11	1	93	95	
Overall, I am completely satisfied with my gender-affirming treatment.								
Treatment Outcome	Strongly disagree	113	26	19	2	23	30	p<0.001
	Mostly disagree	88	37	16	2	34	40	

© 2025 Kaur MN et al. *JAMA Network Open*.

	Slightly disagree	179	48	13	1	46	50	
	Slightly agree	265	54	11	1	52	55	
	Mostly agree	917	68	12	0	67	68	
	Strongly agree	1893	90	12	0	89	90	

UB, upper bound; LB, lower bound; CI, confidence interval

© 2025 Kaur MN et al. *JAMA Network Open*.

eTable 4b. RESULTS FOR HYPOTHESIS-BASED CONSTRUCT VALIDITY TESTING OF THE GENDER-Q SCALES MEASURING SATISFACTION WITH APPEARANCE

Scale<br>All tests significant p<0.001	Overall Question	Extremely dissatisfied					Very dissatisfied					Somewhat dissatisfied					Somewhat satisfied					Very satisfied					Extremely satisfied				
		N	Mean	SD	95% CI		N	Mean	SD	95% CI		N	Mean	SD	95% CI		N	Mean	SD	95% CI		N	Mean	SD	95% CI		N	Mean	SD	95% CI	
					LB	UB				LB	UB				LB	UB				LB	UB				LB	UB				LB	UB
Voice Sound	How your voice sounds overall?	498	24	13	23	25	695	37	5	37	37	972	44	5	44	45	1320	51	5	51	51	1237	62	6	62	62	661	83	14	82	84
Voice Distress	How your voice sounds overall?	494	35	17	34	37	684	45	12	45	46	966	55	11	54	55	1311	64	14	63	65	1227	79	14	78	79	648	88	14	87	90
Face Overall	How your face looks overall?	264	22	12	20	23	408	35	5	35	36	811	43	5	42	43	1500	52	6	52	53	1289	66	7	65	66	598	91	11	90	92
Facial Features	How your face looks overall?	262	36	14	35	38	405	44	7	43	44	806	49	8	48	49	1486	55	9	55	56	1271	66	11	65	67	597	89	13	88	90
Upper Face	How your upper face looks overall?	199	25	15	23	27	321	38	9	37	39	612	47	9	47	48	1298	57	8	57	58	1369	70	8	70	71	951	94	10	93	94
Eyebrows	How your eyebrows look overall?	76	19	16	15	22	140	38	8	37	39	417	45	8	44	46	982	54	7	53	54	1456	71	7	70	71	1158	97	7	96	97
Cheeks	How your cheeks look overall?	114	9	10	7	11	117	27	6	26	29	276	40	6	39	40	433	54	6	53	54	252	72	7	71	73	115	94	10	92	96
Nose	How your nose looks overall?	213	15	13	14	17	266	33	7	32	34	379	43	6	42	43	467	53	6	53	54	316	69	7	68	69	173	94	9	93	95
Nostrils	How your nostrils look overall?	120	12	16	9	15	124	30	6	29	31	279	40	6	40	41	562	54	6	53	54	439	73	7	72	74	229	98	6	97	99
Lips	How your lips look overall?	114	14	13	12	17	170	34	6	33	34	325	43	6	42	44	437	53	5	53	54	275	68	7	68	69	162	90	11	89	92
Chin	How your chin looks overall?	228	9	11	8	11	298	29	6	28	30	455	41	5	41	42	514	54	6	54	55	266	72	7	71	73	136	95	9	94	97
Jaw	How your jawline looks overall?	318	9	11	7	10	382	29	7	28	30	640	41	6	41	42	514	55	6	55	56	249	71	7	70	72	105	93	11	91	95
Facial hair feminization	How your facial hair looks overall?	411	21	12	20	22	223	31	7	31	32	228	39	6	38	39	189	45	6	44	46	142	53	7	52	54	96	64	8	62	66
Facial hair masculinization	How your facial hair looks overall?	102	26	13	23	28	182	37	6	36	38	288	45	5	44	45	558	53	6	53	53	507	65	7	64	65	391	86	13	85	88
Head hair	How your hair looks overall?	224	21	16	19	23	241	39	7	38	39	434	47	6	46	47	906	53	5	53	54	1393	64	7	64	65	1387	90	13	89	90
Body	How your body looks overall?	521	19	13	18	20	687	34	7	33	34	1016	43	6	43	44	1377	52	6	52	53	987	65	8	65	66	362	88	11	87	89
Buttocks	How your buttocks look overall?	288	13	12	11	14	272	32	6	31	32	383	41	5	41	42	423	53	6	52	53	170	68	8	67	70	77	90	12	87	93
Waist	How your waist looks overall?	493	9	11	8	10	513	29	7	29	30	651	42	6	41	42	493	54	7	53	54	193	69	8	68	70	58	91	12	88	94
Breast	Your breasts overall?	138	28	16	26	31	201	43	6	42	44	305	48	5	47	49	526	54	6	53	54	605	63	6	62	63	343	80	13	78	81
Breast Nipples & Areolas	Your nipples and areolas overall?	78	26	19	22	30	112	44	7	43	45	229	48	8	47	50	531	55	7	54	55	637	66	8	66	67	478	91	12	90	92
Vagina	Your vagina overall?	37	15	16	10	20	39	31	10	27	34	93	33	14	31	36	209	43	9	42	44	458	55	10	54	56	386	75	17	73	77
Labia	Your labia overall?	36	23	16	17	28	43	33	10	30	36	93	44	6	43	45	283	50	6	49	51	391	60	9	59	61	294	82	17	80	84
Clitoris	Your clitoris overall?	26	17	17	10	24	49	34	15	29	38	83	43	12	40	45	180	49	11	48	51	378	61	13	60	62	393	83	18	81	85
Chest	How your chest looks overall?	399	16	13	15	18	195	34	10	33	36	142	46	9	45	48	320	59	10	57	60	816	73	12	72	74	975	92	11	91	92
Chest Nipples & Areolas	Your nipples and areolas overall?	245	27	17	25	29	201	41	7	40	42	335	47	8	46	48	553	54	8	53	54	651	66	9	65	66	540	90	12	89	91
Penis	Your penis overall?	12	20	16	10	30	15	35	11	29	41	32	44	7	42	47	73	54	8	52	56	141	63	8	61	64	113	79	14	77	82
Glans	Your glans overall?	14	26	18	16	37	10	34	8	28	40	12	47	7	42	52	48	54	10	51	57	73	63	10	61	66	66	84	15	80	87
Scrotum	Your scrotum overall?	11	20	15	10	30	16	38	7	34	42	27	43	6	41	46	74	51	5	49	52	95	62	8	60	64	83	81	15	77	84
												Dissatisfied					Somewhat satisfied					Very satisfied					Extremely satisfied				

© 2025 Kaur MN et al. *JAMA Network Open*.

Testicular Implants	Your testicular implants overall?	*Dissatisfied categories merged due to sample size	11	23	17	12	34	20	42	7	39	45	23	53	9	49	57	39	77	20	70	83
Perineum	Your perineum overall?		14	23	15	14	32	32	36	11	32	40	56	57	9	55	59	70	88	16	85	92
Erectile Device	Your erectile device overall?		17	36	10	31	41	19	43	9	38	47	24	56	8	53	59	17	73	11	68	79

© 2025 Kaur MN et al. *JAMA Network Open*.

eTable 5. Distribution of Urinary Function Checklist*

Checklist item	Masculine appearance										Feminine appearance									
	Never		Rarely		Sometimes		Often		Always		Never		Rarely		Sometimes		Often		Always	
	n	%	n	%	n	%	n	%	n	%	n	%	n	%	n	%	n	%	n	%
1. …hurts to pee.	246	69.3	62	17.5	27	7.6	14	3.9	6	1.7	908	80.6	177	15.7	35	3.1	4	0.4	2	0.2
2. …interferes with my ability to leave house.	295	82.9	29	8.1	19	5.3	9	2.5	4	1.1	1022	90.7	61	5.4	34	3.0	8	0.7	2	0.2
3. …leak when sleep.	288	80.7	28	7.8	19	5.3	14	3.9	8	2.2	921	81.8	123	10.9	52	4.6	20	1.8	10	0.9
4. … hold my pee when I need to go.	276	77.7	33	9.3	25	7.0	16	4.5	5	1.4	806	71.5	184	16.3	86	7.6	40	3.5	11	1.0
5. …leak when physically active.	291	82.0	32	9.0	21	5.9	3	0.8	8	2.3	955	84.8	101	9.0	49	4.4	16	1.4	5	0.4
6. … takes a long time to pee.	250	70.0	44	12.3	32	9.0	15	4.2	16	4.5	868	77.2	140	12.5	74	6.6	32	2.8	10	0.9
7. …leak when need to pee urgently.	263	73.9	41	11.5	30	8.4	15	4.2	7	2.0	731	65.2	177	15.8	134	12.0	54	4.8	25	2.2
8. …pee comes out like a spray.	210	59.3	52	14.7	57	16.1	20	5.6	15	4.2	412	36.6	235	20.9	263	23.4	156	13.9	60	5.3
9. …leak when cough or sneeze.	289	81.4	34	9.6	21	5.9	7	2.0	4	1.1	898	80.1	137	12.2	68	6.1	16	1.4	2	0.2
10. …hard time starting to pee.	255	72.0	53	15.0	32	9.0	10	2.8	4	1.1	889	79.1	146	13.0	63	5.6	20	1.8	6	0.5
11. …trouble emptying my bladder.	244	68.5	47	13.2	38	10.7	18	5.1	9	2.5	772	68.7	184	16.4	106	9.4	45	4.0	17	1.5
12. …urge interferes with sleep.	243	68.3	46	12.9	43	12.1	12	3.4	12	3.4	713	63.3	188	16.7	139	12.3	58	5.1	29	2.6
13. …need to push to get pee out.	225	63.4	56	15.8	37	10.4	20	5.6	17	4.8	847	75.2	175	15.5	78	6.9	17	1.5	9	0.8
14. …more pee that dribbles out.	102	28.7	49	13.8	68	19.2	60	16.9	76	21.4	544	48.4	264	23.5	192	17.1	92	8.2	33	2.9
15. …pee more often than I think I should.	226	63.7	56	15.8	39	11.0	19	5.4	15	4.2	690	61.4	205	18.2	134	11.9	66	5.9	29	2.6

*The GENDER-Q scales are copyright of McMaster University and Brigham and Women's Hospital (© 2024, McMaster University and Brigham and Women's Hospital). The GENDER-Q must not be copied, distributed, or used in any way without the prior consent of McMaster University.

eTable 6. Distribution OF Surgery, Adverse Effect Checklist*

Checklist item	Face										Chest/Breast area										Genitals									
	Not at all concerned		A little concerned		Somewhat concerned		Very concerned		Extremely concerned		Not at all concerned		A little concerned		Somewhat concerned		Very concerned		Extremely concerned		Not at all concerned		A little concerned		Somewhat concerned		Very concerned		Extremely concerned	
	n	%	n	%	n	%	n	%	n	%	n	%	n	%	n	%	n	%	n	%	n	%	n	%	n	%	n	%	n	%
1. Trouble urinating																					1154	77.1	181	12.1	90	6.0%	37	2.5	35	2.3
2. Trouble concentrating	230	87.5	17	6.5	10	3.8	5	1.9	1	0.4	1476	86.5	109	6.4	77	4.5	28	1.6	17	1.0	1257	84.4	121	8.1	61	4.1	29	1.9	21	1.4
3. Stiffness	212	80.9	26	9.9	14	5.3	10	3.8	0	0.0	1323	77.6	241	14.1	99	5.8	36	2.1	6	0.4	1252	84.4	160	10.8	49	3.3	17	1.1	6	0.4
4. Tenderness	195	74.7	42	16.1	14	5.4	10	3.8	0	0.0	1156	67.8	377	22.1	122	7.2	38	2.2	11	0.6	1122	75.5	227	15.3	95	6.4	27	1.8	15	1.0
5. Burning sensation	243	92.7	9	3.4	7	2.7	3	1.1	0	0.0	1556	91.3	87	5.1	39	2.3	17	1.0	5	0.3	1308	87.8	102	6.9	44	3.0	24	1.6	11	0.7
6. Odor or smell	243	93.1	5	1.9	9	3.4	2	0.8	2	0.8	1643	96.3	34	2.0	19	1.1	9	0.5	1	0.1	1002	67.0	319	21.3	115	7.7	38	2.5	21	1.4
7. Bleeding	238	90.5	15	5.7	8	3.0	2	0.8	0	0.0	1627	95.4	46	2.7	21	1.2	5	0.3	7	0.4	1289	86.4	114	7.6	56	3.8	21	1.4	12	0.8
8. Constipation	245	93.5	8	3.1	7	2.7	2	0.8	0	0.0	1592	93.5	61	3.6	29	1.7	15	0.9	5	0.3	1317	88.4	96	6.4	46	3.1	19	1.3	12	0.8
9. Throbbing feeling	234	89.7	13	5.0	10	3.8	3	1.1	1	0.4	1523	89.6	119	7.0	44	2.6	10	0.6	4	0.2	1345	90.2	104	7.0	21	1.4	13	0.9	8	0.5
10. Bruising	238	90.8	14	5.3	6	2.3	4	1.5	0	0.0	1596	93.8	60	3.5	30	1.8	15	0.9	1	0.1	1400	94.0	58	3.9	19	1.3	9	0.6	4	0.3
11. Pain when you rest	237	90.1	9	3.4	7	2.7	9	3.4	1	0.4	1480	87.0	136	8.0	53	3.1	26	1.5	7	0.4	1320	88.6	116	7.8	29	1.9	16	1.1	9	0.6
12. Discharge (eg, blood, fluid)	245	93.2	8	3.0	7	2.7	2	0.8	1	0.4	1602	94.0	58	3.4	27	1.6	13	0.8	4	0.2	1202	80.7	176	11.8	70	4.7	29	1.9	13	0.9
13. Excess perspiration (sweating)	246	93.5	6	2.3	7	2.7	3	1.1	1	0.4	1540	90.5	89	5.2	47	2.8	18	1.1	7	0.4	1324	88.8	93	6.2	52	3.5	13	0.9	9	0.6
14. Trouble sleeping	226	86.3	15	5.7	15	5.7	5	1.9	1	0.4	1432	84.1	154	9.0	70	4.1	31	1.8	15	0.9	1245	83.6	129	8.7	56	3.8	39	2.6	21	1.4
15. Tingling (pins and needles feeling)	203	77.5	36	13.7	18	6.9	5	1.9	0	0.0	1304	76.7	268	15.8	101	5.9	18	1.1	9	0.5	1280	85.9	132	8.9	54	3.6	11	0.7	13	0.9
16. Lack of feeling (numb)	163	62.0	58	22.1	28	10.6	13	4.9	1	0.4	901	52.8	518	30.4	200	11.7	57	3.3	29	1.7	1078	72.3	232	15.5	101	6.8	49	3.3	32	2.1
17. Feeling depressed or anxious	199	76.0	36	13.7	15	5.7	7	2.7	5	1.9	1349	79.2	202	11.9	95	5.6	36	2.1	22	1.3	1020	68.5	227	15.2	127	8.5	67	4.5	49	3.3
18. Feeling of pressure	224	85.5	22	8.4	10	3.8	3	1.1	3	1.1	1502	88.2	116	6.8	60	3.5	20	1.2	4	0.2	1279	85.7	126	8.4	54	3.6	18	1.2	15	1.0

© 2025 Kaur MN et al. *JAMA Network Open*.

19. Swelling or puffiness	211	80.5	25	9.5	17	6.5	7	2.7	2	0.8	1488	87.3	133	7.8	52	3.1	23	1.3	8	0.5	1261	84.4	135	9.0	60	4.0	25	1.7	13	0.9
20. Soreness	216	82.4	24	9.2	15	5.7	4	1.5	3	1.1	1342	79.0	241	14.2	76	4.5	28	1.6	12	0.7	1148	76.8	210	14.1	85	5.7	34	2.3	17	1.1
21. Pain when you move around	233	88.9	15	5.7	7	2.7	6	2.3	1	0.4	1451	85.4	152	8.9	55	3.2	31	1.8	11	0.6	1245	83.5	166	11.1	43	2.9	21	1.4	16	1.1
22. Pulling sensation	230	87.5	18	6.8	9	3.4	6	2.3	0	0.0	1259	73.9	305	17.9	94	5.5	36	2.1	9	0.5	1253	84.0	156	10.5	56	3.8	13	0.9	14	0.9
23. Aching feeling	233	88.6	14	5.3	8	3.0	6	2.3	2	0.8	1431	84.2	177	10.4	61	3.6	25	1.5	6	0.4	1262	84.7	135	9.1	58	3.9	21	1.4	14	0.9
24. Feeling tired	219	83.3	27	10.3	9	3.4	5	1.9	3	1.1	1398	82.2	150	8.8	98	5.8	33	1.9	22	1.3	1115	74.7	188	12.6	114	7.6	53	3.6	22	1.5
25. Itchiness	213	81.0	35	13.3	7	2.7	6	2.3	2	0.8	1346	79.1	230	13.5	91	5.3	25	1.5	9	0.5	1234	82.9	182	12.2	49	3.3	14	0.9	10	0.7
26. Tightness	212	81.5	29	11.2	14	5.4	4	1.5	1	0.4	1283	75.2	293	17.2	89	5.2	33	1.9	9	0.5	1150	77.1	198	13.3	92	6.2	31	2.1	21	1.4
27. Discomfort	215	82.4	25	9.6	13	5.0	4	1.5	4	1.5	1335	78.5	247	14.5	76	4.5	35	2.1	8	0.5	1105	74.0	242	16.2	86	5.8	39	2.6	21	1.4
28. Feeling light-headed	241	91.6	13	4.9	3	1.1	6	2.3	0	0.0	1582	93.1	62	3.6	37	2.2	12	0.7	6	0.4	1338	89.7	91	6.1	37	2.5	16	1.1	9	0.6
29. Trouble eating or drinking	238	90.5	8	3.0	12	4.6	3	1.1	2	0.8	NA	NA	NA	NA	NA	NA	NA	NA	NA	NA	NA	NA	NA	NA	NA	NA	NA	NA	NA	NA
30. Trouble breathing through your nose	215	82.4	28	10.7	12	4.6	4	1.5	2	0.8	NA	NA	NA	NA	NA	NA	NA	NA	NA	NA	NA	NA	NA	NA	NA	NA	NA	NA	NA	NA

*The GENDER-Q scales are copyright of McMaster University and Brigham and Women's Hospital (© 2024, McMaster University and Brigham and Women's Hospital). The GENDER-Q must not be copied, distributed, or used in any way without the prior consent of McMaster University.

eTable 7. Mean Scores and Key Demographic Characteristics for Scales

Scale	Rasch N	Scale score		Age				Gender identity						Preferred appearance outcome of care – overall						% Missing	Sample who completed in survey
		Mean	SD	Mean	SD	Min	Max	Man		Woman		Other		Masculinization		Feminization		Other			
								n	%	n	%	n	%	n	%	n	%	n	%		
HEALTH-RELATED QUALITY OF LIFE																					
Body Image	4525	57	24	33	12	18	83	1526	34	1610	36	1389	31	2226	49	1806	40	491	11	1	Core
Gender Dysphoria	4519	62	20	33	12	18	83	1514	34	1626	36	1379	31	2210	49	1821	40	485	11	3	Core
Social Acceptance	4621	73	17	33	12	18	83	1548	34	1659	36	1414	31	2265	49	1857	40	496	11	2	Core
Psychological Distress	4454	65	21	33	12	18	83	1499	34	1593	36	1362	31	2189	49	1787	40	476	11	1	Core
Psychological Well-Being	4557	62	20	33	12	18	83	1532	34	1626	36	1399	31	2239	49	1824	40	492	11	2	Core
Treatment Outcome	3469	75	22	34	12	18	83	1263	36	1305	38	901	26	1793	52	1428	41	246	7	2	Had surgery on face, top, bottom - asked to think of most recent
SEXUAL																					
Sexual Well-Being	3898	60	17	32	11	18	76	1369	35	1316	34	1213	31	2007	52	1480	38	410	11	4	Engaged in sexual activity in the last year; and if had bottom surgery - have engaged in sex after surgery
Orgasm	1470	65	19	37	12	18	76	463	32	790	54	217	15	558	38	867	59	44	3	3	
URINATION																					
Urinary Function - CHECKLIST																					Wanted/had bottom surgery, report trouble urinating and do not currently have a catheter
Urinary Catheter	215	50	21	36	13	19	76	52	24	145	67	18	8	58	27	156	73	1	1	3	Had bottom surgery in the last 6 months and had a catheter
GENDER PRACTICES																					
Binding - Well-Being	367	65	19	26	8	18	64	207	56	0	0	160	44	314	86	0	0	53	14	1	Reported binding in the past week
Binding - Chest Symptoms	367	69	18	26	8	18	64	208	57	0	0	159	43	314	86	0	0	53	14	1	
Binding – Skin Symptoms	366	72	19	26	8	18	64	207	57	0	0	159	43	313	86	0	0	53	15	1	
Tucking - Symptoms	306	77	18	36	13	18	73	0	0	264	86	42	14	0	0	295	96	11	4	6	Reported tucking in the past week
VOICE																					
Sound	5415	52	18	33	12	18	83	1822	34	1991	37	1602	30	2648	49	2221	41	545	10	5	Core
Distress	5367	64	21	33	12	18	83	1806	34	1975	37	1586	30	2627	49	2203	41	536	10	2	Core
HAIR																					
Face - Feminization	1584	36	16	38	14	18	81	1	0	1332	84	251	16	0	0	1584	100	0	0	5	All, except those who did not grow facial hair
Face - Masculinization	2043	58	19	30	10	18	71	1443	71	1	0	599	29	2043	100	0	0	0	0	7	
Head	1703	52	18	36	13	18	81	455	27	915	54	333	20	597	35	1008	59	97	6	3	All, except those who were bald
FACE & NECK																					
Face Overall	4898	56	19	33	12	18	83	1637	33	1790	37	1471	30	2392	49	1995	41	508	10	4	Core

© 2025 Kaur MN et al. *JAMA Network Open*.

Facial Features	4854	59	17	33	12	18	83	1622	33	1776	37	1456	30	2364	49	1981	41	506	10	2	Core
Upper Face	1582	52	20	36	13	18	81	149	9	1173	74	260	16	201	13	1295	82	84	5	3	Core
Eyebrows	1564	59	21	36	13	18	81	146	9	1161	74	257	16	197	13	1281	82	84	5	2	Core
Cheeks	866	51	23	38	14	18	81	85	10	634	73	147	17	116	13	696	81	52	6	3	Treatment/Surgery status - unsure, want, had, had & need revisions
Nose	1438	47	22	37	13	18	81	141	10	1012	70	285	20	202	14	1116	78	119	8	3	
Nostrils	1386	56	24	37	13	18	81	137	10	974	70	275	20	197	14	1074	78	115	8	2	
Lips	1050	49	20	39	14	18	81	92	9	766	73	192	18	134	13	835	80	80	8	4	
Chin	1496	45	24	36	13	18	81	213	14	1006	67	277	19	290	19	1113	75	91	6	2	
Jawline	1905	42	22	34	13	18	81	387	20	1031	54	487	26	568	30	1155	61	180	10	3	
Adam's Apple	1146	69	26	37	13	18	81	1	0	1012	88	133	12	0	0	1120	98	26	2	3	
BODY																					
Body	4973	50	19	33	12	18	83	1669	34	1798	36	1506	30	2441	49	2010	40	520	11	3	Core
Buttocks	1406	42	21	36	13	18	81	294	21	756	54	356	25	436	31	839	60	131	9	3	Treatment/Surgery status - unsure, want, had, had & need revisions
Waist	2225	37	21	34	13	18	77	643	29	945	43	637	29	965	43	1048	47	212	10	2	
BREASTS																					
Breasts	2131	57	16	38	14	18	83	1	0	1855	87	275	13	0	0	2129	100	2	0	4	Surgery status - unsure, want, had, had & need revisions OR prefer fem chest and have bra cup size at least AA
Nipples & Areolas	2071	64	20	38	14	18	83	1	0	1804	87	266	13	0	0	2069	100	2	0	3	
GENITAL FEMINIZATION																					
Vagina	1236	56	21	40	14	18	83	0	0	1133	92	103	8	0	0	1221	99	15	1	6	Had feminizing bottom surgery more than 2 weeks ago
Labia	1152	60	19	40	14	18	83	0	0	1061	92	91	8	0	0	1138	99	14	1	6	Had feminizing bottom surgery more than 2 weeks ago & have inner and/or outer labia
Clitoris	1118	63	23	40	14	18	83	0	0	1029	92	89	8	0	0	1107	99	11	1	2	Had feminizing bottom surgery more than 2 weeks ago & have clitoris
Dilation	930	60	20	39	13	18	77	0	0	860	93	70	8	0	0	923	99	7	1	1	Had feminizing bottom surgery more than 2 weeks ago & use a dilator
CHEST																					
Chest	2857	66	28	29	10	18	74	1741	61	4	0	1112	39	2762	97	0	0	95	3	3	Surgery status - want, had, had & need revisions
Scars	1927	79	18	31	10	18	71	1289	67	4	0	634	33	1878	98	0	0	49	3	3	Had chest surgery more than 6 months ago
Nipples & Areolas	2535	60	22	29	10	18	74	1619	64	3	0	913	36	2479	98	0	0	56	2	2	Surgery status - want, had, had & need revisions; and if had surgery had a least one nipple
GENITAL MASCULINIZATION																					
Penis	391	62	18	36	12	19	69	340	87	0	0	51	13	381	97	0	0	10	3	4	Had masculinizing bottom surgery more than 2 weeks ago
Penis Sensation	281	55	27	36	12	19	69	333	87	0	0	49	13	374	98	0	0	8	2	8	
Glans	223	63	20	35	11	19	68	202	91	0	0	21	9	219	98	0	0	4	2	4	Had surgery to create a glans more than 2 weeks ago
Scrotum	310	60	19	36	12	19	68	282	91	0	0	28	9	307	99	0	0	3	1	5	Had surgery to create a scrotum more than 2 weeks ago

© 2025 Kaur MN et al. *JAMA Network Open*.

Perineum	174	63	27	35	12	19	68	158	91	0	0	16	9	173	99	0	0	1	1	5	Had surgery to create a perineum more than 2 weeks ago
Donor Site – Forearm or Thigh	252	65	22	35	11	19	69	227	90	0	0	25	10	248	98	0	0	4	2	0	Donor site located on forearm, thigh, lower leg or back, and phalloplasty not in last 2 weeks
Donor Site – Adverse Effects	251	78	18	35	11	19	69	226	90	0	0	25	10	247	98	0	0	4	2	5	Donor site located on forearm, thigh, lower leg or back, and phalloplasty not in last 2 weeks
Testicular Implants	95	57	24	39	14	19	67	90	95	0	0	5	5	95	100	0	0	0	0	4	Have testicular implants
Erectile Device	78	77	52	37	11	21	66	73	94	0	0	5	6	76	97	0	0	2	3	10	Have an erectile device
EXPERIENCE OF CARE																					
Health Professional	3017	87	18	33	12	18	81	1026	34	1167	39	824	27	1481	49	1312	44	223	7	4	Visited a healthcare professional in the last 6 months for gender-affirming care
Clinic	2333	87	18	34	12	18	81	800	34	910	39	623	27	1153	49	1018	44	162	7	3	Visited a healthcare professional in the last 6 months and physically attended a clinic or office with employees (office staff)
Surgery - Information	530	76	21	32	12	18	76	169	32	190	36	171	32	275	52	205	39	50	9	3	Had top or bottom surgery in the last 6 months
Surgery - Adverse Effects - CHECKLIST																					Had facial, top or bottom surgery
Surgery – Return to Activity	594	85	20	32	12	18	76	190	32	212	36	192	32	310	52	227	38	57	10	3	Had top or bottom surgery in the last 6 months

© 2025 Kaur MN et al. *JAMA Network Open*.
