# Supplementary material for: Development and Assessment of a Patient-Reported Outcome Instrument for Gender-Affirming Care
Source: JAMA Netw Open. 2025 Apr 18;8(4):e254708. doi: 10.1001/jamanetworkopen.2025.4708 (PMC12008761; doi:10.1001/jamanetworkopen.2025.4708)
Supplement: Supplement 2. — Data Sharing Statement [file jamanetwopen-e254708-s002.pdf]

Data Sharing Statement

Kaur. Development and Assessment of a Patient-Reported Outcome Instrument for Gender-Affirming Care. *JAMA Netw Open*. Published April 18, 2025.
doi:10.1001/jamanetworkopen.2025.4708

Data
Data available: No
